# The COVID-19 Threat: An Opportunity to Rethink the European Economic Constitution and European Private Law

**DOI:** 10.1017/err.2020.42

**Published:** 2020-04-23

**Authors:** Hans-W. MICKLITZ

**Affiliations:** * University of Helsinki, Finland and European University Institute, Robert Schuman Centre for Advanced Studies, Italy; email: Hans.Micklitz@eui.eu.

## I. Crisis as chance: a window of opportunity

Not even five years ago, Mario Draghi proclaimed: “Within our mandate, the [European Central Bank] is ready to do whatever it takes to preserve the euro”.^1^ In Carl Schmittian language, the then-president of the European Central Bank seized on the “state of emergency”^2^ to constitute a new European Economic Constitution based on the premise of financial stability. What remains to be remembered is that the key actor was a European institution, not a national one, an institution with a “constitutional” mandate under the Maastricht Treaty. Draghi’s short sentence appeased the financial markets and by and large the Member States followed the credo and established the Banking Union.^3^

The crisis to be managed had a limited focus – monetary and fiscal policy – with broad implications for the economy and society in the European Union (EU) and the Member States, however. The COVID-19 threat is different. The “state of emergency” requires management of a catastrophe that shatters societies (the lives of the people, nation states) and the capacities of national healthcare systems to handle the fatal threat, which shatters the EU with its lack of competences beyond the Internal Market. Last but not least, the COVID-19 crisis demonstrates the vulnerability of a globalised economy, where medical devices are produced in global supply chains. In contrast to the euro crisis, the European legal order does not provide for institutions that are empowered to manage crises and that have the means and remedies to fight the COVID-19 threat. It suffices to imagine the potential effects of a Draghi-like declaration by the current president of the European Commission.

Under the COVID-19 threat, the key actors are the nation states, more accurately national governments and their political leaders. What we can observe is a revival of the “political”^4^ through the Member States. This is exactly what critiques of European neoliberalism were asking for.^5^ The nation states are taking action within the boundaries of their national democratic constitutions and the EU legal order.^6^ They act through legislative packages to save the lives of their citizens and EU citizens in border regions through the establishment of a safety net for the economy, for employees and for companies. The COVID-19 threat dictates the order of assistance – health first, then money; society first, the economy second. This order demonstrates and explains the weakness of the EU as a quasi-state with a quasi-constitutional legal order built around enumerated powers and the still-remaining foundational powers of the Member States as the guardians of the Treaty in terms of the management of societal crises. The EU has no competence-competence.

The COVID-19 threat offers legal scholars a unique opportunity to seriously think about the legal order that should govern the society we want to live in and an economy that serves the expectations of people in the post-COVID-19 world.^7^ The COVID-19 threat has opened a window of opportunity for transgressing boundaries, for thinking the unthinkable: a fundamental revision of the European Economic Constitution and therewith European private law. The window will not remain open for long. The longer the shutdown lasts, the stronger the voices will be to return to normal, to bring people back to work, to get the economy going again; and the more the political debate around crisis management turns to its economic implications, the easier it will become for the EU to gain ground again and to integrate national crisis management into the European legal order and into the set of competences the EU has and that allows it to manage the economic effects of the COVID-19 threat. Depending on the depth and length of the expected recession, one might even think of a much more radical scenario, one where the Member States use the EU as a catalyst to promote a hard-core version of economic neoliberalism where the social and environmental achievements of recent decades are blown away as barriers in order to relaunch the economy.

The revival of economics will come. What is therefore needed here and now is a rethinking of the European Economic Constitution and European private law. In the potential euphoria surrounding new perspectives, it should not be forgotten that the current crisis management by the EU Member States relies on the full functioning of key economic sectors – food production and supply, banking, healthcare, transport, the Internet, energy – more often than not provided by multinational, transnational corporations, national incumbents and supermarket chains. The Internal Market has not been suspended, at least not with regards to the cross-border trade in goods and services. What is mainly suspended is personal freedom – in EU language, the free movement of persons and non-essential business activities in areas where small and medium-sized companies dominate. There will be lessons to learn as to who will benefit from the crisis – Member State politics, national governments, multinationals, online business, transport of goods – and who will suffer: the EU as an institution, the European legal order based on the four freedoms and competition, national parliaments, small and medium-sized companies and non-essential economic sectors. A stocktaking will be needed on what remains of the European legal order after COVID-19 and whether and to what extent the state of emergency the Member States have proclaimed could be brought into line with EU law. This is the kind of clean-up that lawyers are accustomed to doing as a repair service for society and the economy.

## II. Rethinking

What, then, does rethinking – thinking the unthinkable – imply? Thinking the unthinkable does not relate to Krastev’s “After Europe”^8^ – the dissolution of the EU overnight. The majority of European legal scholars might in one way or the other sympathise with Baquero Cruz,^9^ with the deeper historical origins of the “Law after Auschwitz” (the heading of Cruz’s chapter 2): its peace through trade mission, a rejection of the return to the nation state and the political and theoretical opportunity for a serious and substantial reform of the EU. In rethinking, there is no need to start from scratch. From the 1990s onwards, growing critical legal scholarship has accompanied the eastern enlargement of the EU and the feasibility of a European Constitution and of a European Civil Code. By and large, the critique has fallen on deaf ears: the eastern enlargement was pushed through against warnings about the impact of the “one-size-fits-all” approach on the former socialist economies, let alone the lack of democratic institutional structures and a society that subscribes to liberal democratic values. The failed European Constitution was saved in the Lisbon Treaty, and the failed European Civil Code was broken down into a bunch of regulations and directives that breathe the same spirit. In the COVID-19 threat, the criticism appears in a different light, not as a laborious attempt to change the direction of European politics, but as a possible scenario for a new beginning. In what follows, I would like to tie three strands of discussion together: the COVID-19 threat, the UN Sustainable Development Goals and the circular economy, as well as the digital economy and society. Only such a holistic perspective opens up pathways for a new European Economic Constitution and a new European private law post-COVID-19.

### 1. Society

The COVID-19 threat has laid bare the missing dimension of all of the European Economic Constitution and European private law rhetoric of the last 20 years: the inclusion of the societal dimension of the European integration process into the European legal order. The exclusion of the social impact of the Internal Market on national societies and on an infant European society builds the first strand of critique that unites philosophers and legal theorists. In his letter to P. van Parijs, Rawls asks whether “meaningless consumerism” could be a legitimate aim for the EU and for a European society.^10^ Davies and Taguri highlight the societal and cultural implications of ever-increasing consumer choice in the Internal Market.^11^ The one-sided promotion of online business, which empties our cities and transforms physical communication into online orders, is just one visible expression of what is excluded from the toolbox of European law – not only to investigate potential gains in economic growth through increased efficiency and increased effectiveness of political objectives through fully harmonising European private law rules, but also to seriously look into their impacts on society.

What can law contribute to the building of a national society? The eastern enlargement seems to teach the lesson that there are limits to the role of law in post-communist states, where society had been suppressed for over 40 years. This does not mean that European law cannot play a much larger role in the making of a European society. The non-existence of a fully-fledged European state opens up more – not fewer – opportunities for the law to enable society-building.^12^ This implies understanding the law of the Internal Market as the law of the Internal Market *society*.^13^ The addressee of such a legal order is the consumer citizen, the worker citizen, the employer citizen. Such an understanding breaks down the fragmentation of the legal person in the EU^14^ and establishes a common umbrella for status-related European laws (non-discrimination, labour and consumer law). Whether it is feasible to combine citizenship with economic rights is subject to controversy.^15^ In an admittedly optimistic reading, I have tried to formulate out of the bits and pieces of European private law *de lege lata* a tripartite distinction between universal services, the law of the labour market *society* and consumer market *society* and *societal* private law.^16^

### 2. Sustainability

The second potential for rewriting the European Economic Constitution is the ever-louder call for sustainability, which reached the EU through adoption of the circular economy in 2015. The COVID-19 threat provides the earth with breathing space. The Juncker Commission has brought to a “successful” end a whole range of regulations and directives that cover broad fields of economic and private law. These newly adopted legislative measures follow the Internal Market philosophy, the rationality of market efficiency piggy-backing on social regulations on consumer and environmental protection. They are united in that sustainability and the circular economy are downgraded to a few window-dressing references in the recitals without any conceptual rethinking, let alone re-conceptualising. Sustainability goals translated into the circular economy are kept strictly separated from economic (Internal Market) and private law. There is no attempt to initiate a debate that the UN Sustainable Development Goals and the circular economy, if taken seriously, might require a different economic constitution and a different private law. What is urgently needed is a serious stocktaking of the state of affairs in terms of European private law in the light of the 17 Sustainable Development Goals that highlight the dark side of one-sided rules that protect employees and consumers without taking their counterproductive effects on the environment into account.^17^

In his “many constitutions”, K. Tuori^18^ distinguishes between the framing of juridical and political constitutions and the sectoral constitutions: micro- and macro-economic, social and security constitutions. Neither he nor von Bogdandy and Bast^19^ in their compendium mention the possible existence of an environmental/sustainable constitution. The bits and pieces enshrined in Article 3 of the Treaty on the Functioning of the European Union (TFEU) and Article 191 TFEU render it difficult if not impossible to deduce concrete consequences of the positioning of the environment in relation to the micro- and macro-economic constitution.^20^ Here is the place for a political commitment by the Member States to give shape to a new European economic constitution. The COVID-19 threat is supposed to strengthen the transformation of the nation state into a steering state – “l’État Providence” in the format of the precautionary state.^21^ The EU Member States will have to make sure that the healthcare sector will never again suffer from shortages in life-saving medical devices and pharmaceuticals and that essential industries that are indispensable for fighting the *next* pandemic threat are located within the EU if not on national territory. The transformation will affect the European Economic Constitution and the interplay between private entrepreneurship and de facto nationalised industrial sectors. The huge safety net that the Member States are currently building with taxpayers’ money is the means through which the EU Member States intervene in the economy. The very same means could and should be understood as an opportunity to build a sustainable economy that deserves its name. Financial aid could be bound to sustainability.

### 3. Digitisation

The overall assumption is that the COVID-19 threat will boost the digitisation of society and the economy in personal and business communication, in education at schools and universities and in online and offline business. The private law of contracts and private regulation through contracts, codes and standards beyond the nation state enables and promotes digitisation. The EU is at the forefront in setting legal standards through the General Data Protection Regulation, the Copyright Directive, the Platform-to-Business Regulation and, last but not least, through the Directive on Digital Content. The rationale is again to promote economic growth in the Internal (digital) Market through digitisation. The bits and pieces are pillars of an emerging European digital constitution in which the proclaimed human-centred artificial intelligence risks falling by the wayside. The post-COVID-19 wonderland should not be entered without a deeper understanding of the political economy behind digitisation.^22^ Digitisation will be used ever more strongly to consider if, how and to what extent algorithms, machine learning techniques and neuronal nets can reduce the social costs of labour, which will have a huge impact on our societies.^23^ The COVID-19 threat will accelerate and legitimise the digital transformation of society and the economy. However, the very same crisis must be seized as an opportunity to make sure that technology serves, in the words of Brownsword, “the anterior conditions for *humans* to exist and for them to function as a *community of agents*”.^24^ In the scholarly debate on digitisation and the impact of economic and private law, there seems to be agreement that *red lines* are needed that cannot be crossed by anyone and that should not be submitted to a balancing test.^25^ The digital constitution is supposed to set benchmarks for all sorts of European, national and private regulation that governs private relations in the digital society and economy.

Who is the addressee? Can the EU take over the proclaimed stewardship responsibility? Zuboff stressed a move towards surveillance capitalism;^26^ the COVID-19 threat presents the risk of a surveillance state.^27^ It is certainly not enough to expect red lines to be established by states or by the EU. Much will depend on pressure from civil society and from critical legal scholarship to develop models that could be translated into legislative programmes on whether the *lex digitalis* can be turned into a joint exercise of a transnational community.^28^

### 4. Society, sustainability and digitisation

This window of opportunity can only be fully exploited if the three strands of discussion are brought together in the design of a post-COVID-19 European Economic Constitution and European private law. The 2020 working programme of the European Commission, adopted in January 2020, aims at bringing sustainability and digitisation together, without stressing the need, however, to involve national societies and the infant European society. The COVID-19 threat provides critical legal scholarship with a unique opportunity, despite all of the current and future uncertainties and speculations, to move far beyond the mainstream understanding of the economic constitution and private law.

## Footnotes

1 <https://www.ecb.europa.eu/press/key/date/2012/html/sp120726.en.html>.

2 C Schmitt, *Politische Theologie. Vier Kapitel zur Lehre von der Souveränität*, 1922, C Schmitt, *Political Theology: Four Chapters on the Concept of Sovereignty* [1922] (George Schwab trans, Chicago, University of Chicago Press 2010) pp 5–35; and in particular G Agamben, *State of Exception* (Chicago, Chicago University Press 2015).

3 Contributions in S. Grundmann and H-W Micklitz (eds), *The European Banking Union and Constitution: Beacon for Advanced Integration or Death-Knell for Democracy* (Oxford, Hart Publishing 2018).

4 S Bartolino, *The Political* (London, ECPR Press and Rowman & Littlefield International 2018).

5 Most prominently, W Streeck, *How Will Capitalism End? Essays on a Failing System* (New York, Verso 2016); D Kochenov, G de Búrca and A Williams (eds), *Europe’s Justice Deficit?* (Oxford, Hart Publishing 2015).

6 A detailed analysis will certainly reveal a more nuanced picture.

7 M Horx, Die Welt nach Corona <https://www.horx.com/48-die-welt-nach-corona>.

8 I Krastev, *After Europe* (Philadelphia, PA, University of Pennsylvania Press 2017).

9 J Baquero Cruz, *What’s Left of the Law of Integration? Decay and Resistance in European Union Law* (Oxford, Oxford University Press 2018).

10 J Rawls and P Van Parijs, Three letters on *The Law of Peoples* and the European Union, published in *Autour de Rawls*, special issue of *Revue de philosophie économique* (2003) 7, 7–20 <http://www.fondsricoeur.fr/uploads/medias/articles_pr/echanges-rawls-van-parijs.pdf>.

11 G Davies, “Freedom of Contract and the Horizontal Effect of Free Movement Law”, in D Leczykiewicz and S Weatherill (eds), *The Involvement of EU Law in Private Law Relationships* (Oxford, Hart Publishing 2013) pp 53–70; G Taguri, “The Cultural Implications of Market Regulation: Does EU Law Destroy the Texture of National Life?” PhD thesis at Bocconi, Italy, 2018.

12 G Comandé, “The Fifth European Union Freedom. Aggregating Citizenship … around Private Law”, in H-W Micklitz (ed.), *Constitutionalization of European Private Law* (Oxford, Oxford University Press 2014) pp 61–101; K Carr, “Regulating the Periphery: Shaking the Core European Identity Building through the Lens of Contract Law” (2015) EUI Department of Law Research Paper No. 2015/40.

13 S Deakin and F Wilkinson, *The Law of the Labour Market: Industrialization, Employment and Legal Evolution* (Oxford, Oxford University Press 2005).

14 H Collins, “The Revolutionary Trajectory of EU Contract Law towards Post-National law”, in S Worthington, A Robertson and G Virgo (eds.), *Revolution and Evolution in Private Law* (Oxford, Hart Publishing 2017) pp 315–36 distinguishes between instrumentalisation (policy driven), fragmentation (of legal person) and incompleteness.

15 M Everson, “The Legacy of the Market Citizen”, in J Shaw and G More (eds), *New Legal Dynamics of European Union* (Oxford, Clarendon Press 1995) pp 73–90; NN Shuibhne, “The Resilience of EU Market Citizenship” (2010) 47 Common Market Law Review 1597.

16 H-W Micklitz, *The Politics of Justice in European Private Law, Part III: Considerations on the Post-Classical Private Law* (Cambridge, Cambridge University Press 2018).

17 H-W Micklitz, “Squaring the Circle? Reconciling Consumer Law and the Circular Economy”, in B Keirsbilck and E Terryn (eds), *Circular Economy and Consumer Protection* (Cambridge, Intersentia 2019) p 321 and Journal of European Consumer and Market Law (EuCML) (2019) 6, 229–37.

18 K Tuori, *The Many Constitutions of Europe* (Cambridge, Cambridge University Press 2016).

19 A von Bogdandy and J Bast (eds), *Principles of European Constitutional Law* (Oxford, 2^nd^ edn, Hart Publishing 2011).

20 There is an abundant literature on the deficits in the Lisbon Treaty; see A Kiss, “European Environmental Law and the Constitution” (2004) 21 Pace Environmental Law Review 103; P Beyer, “Environment in the Future European Constitution” (2004) 1 Journal for European Environmental & Planning Law 143.

21 J Fischer, former German foreign minister, “Der Staat wird zum Vorsorgestaat, Frankfurter Allgemeine Zeitung”, 31 March 2020; F Ewald, *L’Etat providence* (Paris, Grasset 1986).

22 Y Benkler, “The Role of Technology in Political Economy”, 25 July 2018, Harvard University, Berkman Klein Center <https://cyber.harvard.edu/story/2018-07/role-technology-political-economy>.

23 P Drahos, *The Global Governance of Knowledge: Patent Offices and Their Clients* (Cambridge, New York, Cambridge University Press 2010); R Süsskind, “Legal Tech Book Series: Tomorrow’s Lawyers by Richard Susskind (Part 3)” <https://legal-tech-blog.de/legal-tech-book-series-tomorrows-lawyers-by-richard-susskind-part-3>.

24 R Brownsword, “Responsible Regulation: Prudence, Precaution and Stewardship” (2011) 62 Northern Ireland Legal Quarterly 573.

25 T Wu, “Bigger Brother, Book Review of S. Zuboff, *The Age of Surveillance Capitalism: The Fight for a Human Future at the New Frontier of Power*”, *The New York Review of Books*, 9 April 2020 <https://www.nybooks.com/contributors/tim-wu>; R Brownsword, “Political Disruption, Technological Disruption, and the Future of EU Private Law”, in T Tridimas and M Durovic (eds), *New Directions in European Private Law* (Oxford, Hart Publishing, forthcoming).

26 S Zuboff, “Big Other: Surveillance Capitalism and the Prospects of an Information Civilization” (2015) 30 Journal of Information Technology 75.

27 G Agamben, “Nach Corona: Wir sind nurmehr das nackte Leben”, *Neue Zürcher Zeitung*, 18 March 2020 <https://www.nzz.ch/feuilleton/giorgio-agamben-ueber-das-coronavirus-wie-es-unsere-gesellschaft-veraendert-ld.1547093>.

28 G Teubner, “Societal Constitutionalism: Alternatives to State-Centred Constitutional Theory?”, in C Joerges, I-J Sand and G Teubner (eds), *Transnational Governance and Constitutionalism* (Oxford, Hart Publishing 2004) pp 3–28.

